# 3D Printing Surgical Implants at the clinic: A Experimental Study on Anterior Cruciate Ligament Reconstruction

**DOI:** 10.1038/srep21704

**Published:** 2016-02-15

**Authors:** An Liu, Guang-huai Xue, Miao Sun, Hui-feng Shao, Chi-yuan Ma, Qing Gao, Zhong-ru Gou, Shi-gui Yan, Yan-ming Liu, Yong He

**Affiliations:** 1 Department of Orthopaedic Surgery, Second Affiliated Hospital, School of Medicine, Zhejiang University, Hangzhou 310009, China; 2 State Key Laboratory of Fluid Power and Mechatronic Systems, College of Mechanical Engineering, Zhejiang University, Hangzhou 310027, China; 3 Key Laboratory of 3D Printing Process and Equipment of Zhejiang Province, College of Mechanical Engineering, Zhejiang University, Hangzhou 310027, China; 4 Department of Oral and Maxillofacial Surgery, Second Affiliated Hospital, School of Medicine, Zhejiang University, Hangzhou 310009, China; 5 Zhejiang-California International Nanosystem Institute, Zhejiang University, Hangzhou 310058, China

## Abstract

Desktop three-dimensional (3D) printers (D3DPs) have become a popular tool for fabricating personalized consumer products, favored for low cost, easy operation, and other advantageous qualities. This study focused on the potential for using D3DPs to successfully, rapidly, and economically print customized implants at medical clinics. An experiment was conducted on a D3DP-printed anterior cruciate ligament surgical implant using a rabbit model. A well-defined, orthogonal, porous PLA screw-like scaffold was printed, then coated with hydroxyapatite (HA) to improve its osteoconductivity. As an internal fixation as well as an ideal cell delivery system, the osteogenic scaffold loaded with mesenchymal stem cells (MSCs) were evaluated through both *in vitro* and *in vivo* tests to observe bone-ligament healing via cell therapy. The MSCs suspended in Pluronic F-127 hydrogel on PLA/HA screw-like scaffold showed the highest cell proliferation and osteogenesis *in vitro*. *In vivo* assessment of rabbit anterior cruciate ligament models for 4 and 12 weeks showed that the PLA/HA screw-like scaffold loaded with MSCs suspended in Pluronic F-127 hydrogel exhibited significant bone ingrowth and bone-graft interface formation within the bone tunnel. Overall, the results of this study demonstrate that fabricating surgical implants at the clinic (fab@clinic) with D3DPs can be feasible, effective, and economical.

Ever since Charles Hull first proposed the three-dimensional (3D) printing process in 1986, the technology has developed rapidly and well beyond what originally seemed possible^1^. Nowadays, 3D printing has been utilized successfully in mechanical manufacturing and many areas of scientific research^2^. Many potential uses for 3D printing have emerged within the medical field, not only as far as tissue and organ regeneration research^3^ (blood vessels^4^, ears^5^, bones^6^), but also for customized medical devices such as splints and stents that can be printed in small clinics^7^. There are several factors that limit the use of 3D printers in practice, however; 3D printers necessary for medical applications are specialized or industrial equipment that require unique materials, for example, which drives up production costs and creates a high-level technical demand for skilled operators and specific operational conditions, and the inconvenience of communicating at length between hospitals and factories during the production process delays the length of time between fabrication and application. It was reported that only $11 million was invested in medical applications among the entire 3D printing industry which is worth around $700 million in total^8^. To allow medical professionals and their patients to benefit from 3D printing technologies, and to increase the market share value of 3D medical printing, it is crucial to develop methods that reduce production costs and increase the flexibility, maneuverability, and practicability of the process.

Fused deposition modeling (FDM)^9^, when applied to the 3D printer, creates a desktop 3D printer (D3DP) that can be used at home, in schools, and by small businesses to fabricate customized products cost-effectively. D3DPs cost as little as $500, as opposed to the $15,000–30,000 price range for 3D printers used in academic institutions. If the D3DP can be successfully applied in the medical field, the possibility for cost-effective, personalized devices such as implants or grafts to be fabricated in-clinic is momentous. Doctors and specialists who employ such technology would represent the pioneering edge of the medical field.

In a previous study conducted in our laboratory^10^, we were able to fabricate soft tissue prostheses using a D3DP; the prostheses, which showed smooth surfaces and intricate structures, cost only about $30. The results of this study have considerable implications as far as the future of maxillofacial repair technology. In the present study, we focused on fabricating surgical implants and applying them in operations to demonstrate that a surgeon can indeed customize and fabricate surgical implants his or herself using a D3DP.

Our target operation was an anterior cruciate ligament (ACL) reconstruction using a hamstring tendon graft. This operation requires that the tendon graft within the bone tunnel heal appropriately. Tendon-to-bone tunnel healing occurs through new bone ingrowth that initially forms between the tendon and the bone. With the help of new bone mineralization and maturation, the graft’s biomechanical properties progressively increase – tendon graft healing within the bone tunnel thus mainly depends on the osteointegration of the tendon graft within the bone tunnel^11^.

Bioabsorbable interference screws, made with polymers such as polylactic acid (PLA) and polyglycolic acid (PGA), are commonly used to provide a press fit between bone, graft, and screws initially, which then degrade mainly by hydrolysis as bone union gradually progresses^12^,^13^. According to clinical trials, PLA and PGA screws have been shown to persist *in vivo* for up to 5 years and result in complete resorption at 7 to 10 years^14^,^15^. The relatively slow degradation rate of bioabsorbable screws does not suit the speed of new bone formation, which leads to malformation of new bone around the tendon graft, where only calcified fibrous or fatty tissue replaces the screw in the bone tunnel^15^,^16^.

It has been reported that 3D porous structure is a key point to promote bone ingrowth by providing sufficient growth space. Macropores (200–400 μm) enhance the migration of osteoblasts and osteoprogenitors into the scaffold and facilitate osteoid formation and mineralization^17^. Additionally, interconnected micropores (50–100 μm) can increase vascularization and nutrient diffusion during bone reconstruction^18^. These structures cannot be well-controlled through conventional methods^19^,^20^, but surgeons and specialists can easily and precisely manipulate them using a D3DP.

In this study, common PLA filament, the same as that used for bioabsorbable screws, was applied to D3DP manufacturing of a 3D, porous, screw-like scaffold in-clinic. The scaffold not only could fix the tendon graft, but also could provide adequate space for bone ingrowth around the graft. A simple surface modification was made using hydroxyapatite (HA) on the scaffold in order to enhance osteoconductivity and cellular adhesion^21^, and mesenchymal stem cells (MSCs), known as one of the most optimal cell sources for ACL regeneration due to their high potential for proliferation and collagen production^22^,^23^, were seeded onto the scaffold as cell therapy.

We hypothesize that the 3D-printed, bioabsorbable screw-like scaffold loaded with MSCs can promote tendon graft healing within the bone tunnel by increasing bone ingrowth. We hope that the results of this study will increase the popularity of 3D-printed surgical devices by proving that they can be customized and fabricated feasibly, economically, and successfully in the clinic.

## Methods

### Fabrication and Characterization of PLA Screw-like Scaffold

**Figure 1:**
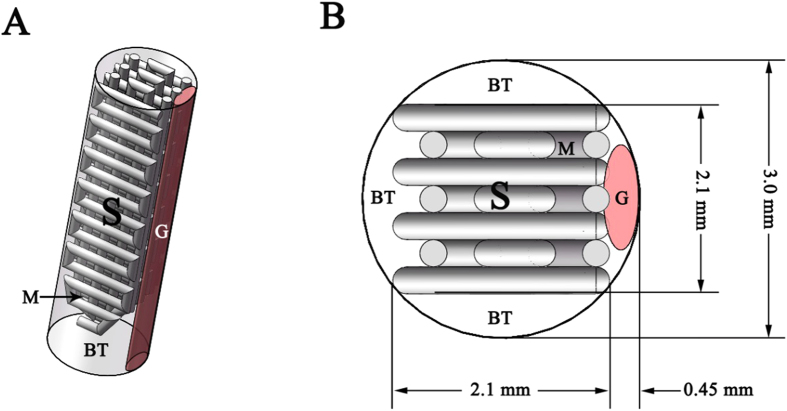
Schematic diagrams of the implant and tendon graft within the bone tunnel in ACL reconstruction. (**A**) The 3D perspective of the bone tunnel in ACL reconstruction. (**B**) The transverse section view of the bone tunnel. (G: Graft; S: Screw-like scaffold; BT: Bone tunnel; M: Macropore).

The PLA screw-like scaffold was designed using Rhinoceros software (ver. 4.0, USA) according to a schematic, actual-size diagram of the implant and tendon graft based on a rabbit ACL reconstruction model (Fig. 1). Its digital dataset was saved as a stereolithography (STL) file. Slice software Slic3r^24^ was used to generate G code for the D3DP (Dot Go 3D Technology Corporation, Xiangtan, China) from the STL file. Melt PLA filament (Shenzhen Esond Technology Co., Ltd) was extruded through a heated metal nozzle (0.4 mm in diameter, moving horizontally and vertically) at 205 °C and deposited onto a receiving station to form the desired scaffolds. The scaffolds were then observed under a scanning electron microscope (SEM) (S-4800, Hitachi, Japan) to measure macropore sizes. The porosity of the scaffolds was determined using the Archimedes method, and the PLA scaffolds were weighed as dry weight (W_1_). The scaffolds were then immersed in a beaker of water and held under vacuum to force the liquid into the pores until no bubbles emerged, then re-weighed under water to determine the suspension weight (W_2_). The scaffolds were then carefully taken out of the beaker and any water on the surface was removed, then they were quickly re-weighed in air to determine the saturated wet weight (W_3_). The final porosity of the scaffolds was calculated via the following equation: porosity (%) = (W_3_ − W_1_)/(W_3_ − W_2_) × 100%. Six specimens were measured in total.

### HA Synthesis and Characterization

HA powders were synthesized by chemical precipitation using Ca(NO_3_)_2_·4 H_2_O and (NH_4_)_2_HPO_4_ as P and Ca precursors, respectively. Ca(NO_3_)_2_·4 H_2_O (Sigma-Aldrich, Australia) was dissolved in distilled water (0.5 mol/L) and adjusted to pH 10.5 with NH_3_·H_2_O. (NH_4_)_2_HPO_4_ (Sigma-Aldrich, USA) was dissolved in distilled water at density of 0.3 mol/L and pH 10.5, then the Ca(NO_3_)_2_ solution was added to the (NH_4_)_2_HPO_4_ solution dropwise. After stirring for 12 h, the precipitate was filtered and subsequently washed three times with distilled water followed by three washing steps with ethanol. The remaining liquid was removed by vacuum filtration, and the precipitate was dried at 80 °C overnight. The resultant powders were calcined at 850 °C for 3 h to obtain HA powders. The calcined HA powders were then ground and sieved through 250 mesh sieves. The crystal morphology of the synthesized HA powder was observed using SEM, and the phase composition of HA was characterized by X-ray diffraction (XRD, Rigaku Co., Japan).

### Surface Modification for PLA/HA Scaffold

Chitosan (CHI) was dissolved in 2% (v/v) acetic acid to obtain CHI solution (1% (w/v)). Sodium alginate (SA) solution (1% (w/v)) was prepared with distilled water. HA powders were added into the CHI and SA solutions, respectively, on a magnetic stirrer plate for 30 min to obtain 4% (w/w) HA/CHI solution and 4% (w/w) HA/SA solution. Sodium hydroxide (NaOH) solution (0.2% (w/w)) was mixed with equal volume of ethanol to prepare NaOH/ethanol solution. The PLA scaffolds were first dipped in the NaOH/ethanol solution under vacuum for 10 min to modify the scaffolds with stable negative charge, then washed twice with distilled water under vacuum, then freeze-dried for 30 min. Next, the scaffolds were immersed in 4% (w/w) HA/CHI solution to force solution into the pores until no bubbles emerged from the scaffolds (10 min) followed by centrifugation (1000 r/min, 5 min). The scaffolds were dried at room temperature for 20 min, then immersed in 4% HA/SA solution under vacuum. The same procedures were repeated for all samples. The PLA/HA scaffolds were then observed with SEM.

### Cell Culture *In Vitro*

MSCs were obtained from bone marrow aspirates of New Zealand Rabbits^25^. Cells of third passage were cultured in Dulbecco’s Modified Eagle Medium (DMEM) supplemented with 10% fetal bovine serum (FBS) (Gibco, USA) in an incubator at 37 °C with 5% CO_2_. Pluronic F-127 was added into complete DMEM to prepare a 30% (w/v) solution at 4 °C. The solution was placed on a magnetic stirrer plate for 24 h to allow complete dissolution, then the solution was filter-sterilized through a 0.22 μm pore size bottle-top filter and stored at 4 °C until use.

After being sterilized with ethylene oxide, the PLA scaffolds and PLA/HA scaffolds were placed into 24-well tissue culture plates (TCPs) and immersed in DMEM with 10% FBS for 2 h, then each was seeded with 1 × 10^5^ MSCs. An equal number of 1 × 10^5^ MSCs suspended in Pluronic F-127 solution were seeded on the PLA/HA scaffolds at 4 °C to ensure the hydrogel penetrated the scaffold, then they were moved to the incubator for gelation.

### Cell Morphology

After 48-hour incubation, samples were washed with phosphate buffer solution (PBS) twice and fixed with 2.5% glutaraldehyde solution for 2 h. The fixed cells were washed with PBS three times and treated with 1% osmium tetroxide for 2 h, then dehydrated in ascending concentrations of ethanol (30, 50, 70, 80, 90, 95, 100 (v/v)) for 5 min, respectively. The samples were then immersed in isoamyl acetate for 20 min, then vacuum-dried at 40 °C for 4 h. The specimens were coated with gold-palladium and dried, then the MSC morphology of each was observed using SEM.

### Cell Viability

MSC viabilities were analyzed with Cell Counting Kit-8 (CCK-8, Dojindo, Japan) assays at 1, 4, and 7 days. DMEM (0.5 mL) containing 10% CCK-8 was added into each well. After 120 min, 100 μL of the abovementioned solution was transferred to a 96-well plate. A microplate reader (Infinite F50, TECAN, Switzerland) was used to measure solution absorbance at 450 nm, and absorbance values were corrected by subtracting the signal of a mixture of 90 μL DMEM and 10 μL CCK-8. Five specimens were prepared for each sample.

### Real-time Polymerase Chain Reaction (PCR) Analysis

Real-time PCR was used to detect the expression of several osteogenetic, differentiation-related marker genes (Col I, OCN, Sp7, and Runx2) at Day 7. Total RNA was extracted using Trizol reagent (Invitrogen) according to the manufacturer’s instructions. NanoDrop 2000c (Thermo Fisher Scientific Inc., USA) was used to determine the total RNA concentration. First-stranded complementary DNAs (cDNAs) were synthesized from 0.5 μg of the isolated RNA by oligo(deoxythymidine) (oligo (dT)) using the DyNamoTM cDNA Synthesis Kit (Fermentas) and used as templates for real-time PCR. The PCR was performed on a final volume of 25 μL containing 1 μL cDNA, 0.5 μL of each primer (forward and reverse), 12.5 μL Power SYBR^®^ Master Mix (2×) (Applied Biosystems, Foster City, CA, USA), and 10.5 μL dd H_2_O with the Bio-Rad Real-time PCR System (Bio-Rad, Hercules, CA, USA), using glyceraldehydes-3-phosphatedehydrogenase (GADPH) as the house-keeping gene for normalization. The forward and reverse primer sequences utilized are listed in Table 1. The conditions of real-time PCR were 95 °C for 1 min, followed by 40 cycles at 95 °C for 10 s and 64 °C for 25 s.

### ACL Reconstruction

**Figure 2:**
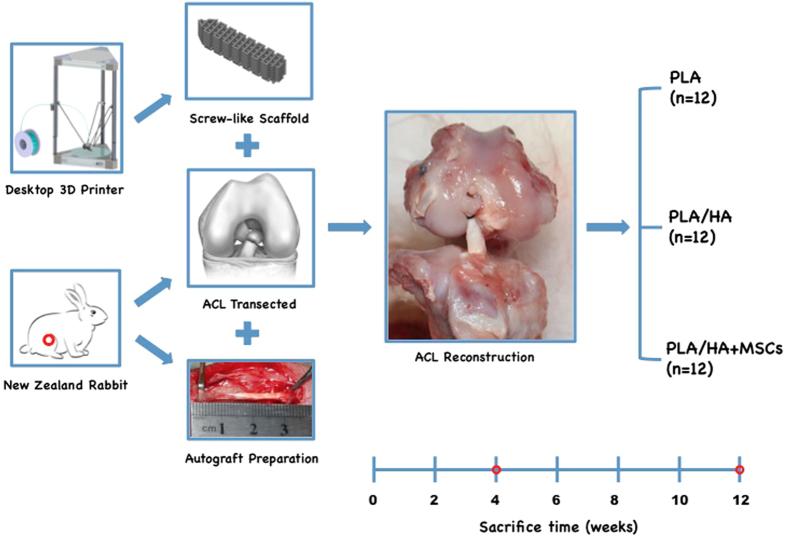
Study design *in vivo*. The screw-like scaffold was designed and fabricated by the D3DP. All rabbits were randomly divided into PLA group (PLA scaffold implantation, n = 12), PLA/HA group (PLA/HA scaffold implantation, n = 12) and MSCs group (PLA/ HA scaffold loaded MSCs, n = 12). Complete sharp transections were established in the anterior cruciate ligament of adult New Zealand male rabbits. The long digital extensor tendon (2 mm in diameter and 3 cm in length) was harvested as tendon graft. The scaffold was pressed into the femoral tunnel to fix the tendon graft. The rabbits were sacrificed at weeks 4, 12 for subsequent analysis.

A total of 36 New Zealand male rabbits weighing 2.5–3.0 kg were utilized in this study according to standard guidelines approved by the Zhejiang University Ethics Committee (ZJU2014-1-05-093). All rabbits were randomly divided into the PLA group (PLA scaffold implantation, n = 12), PLA/HA group (PLA/HA scaffold implantation, n = 12), or MSCs group (PLA/ HA scaffold loaded MSCs, n = 12). Next, 2 × 10^5^ of MSCs suspended in Pluronic F-127 solution were loaded on the PLA/HA screw-like scaffolds at 4 °C and cultured *in vitro* at 37 °C with 5% CO_2_ over 8 h for gelation and cell adhesion before implantation. The animals were subjected to general anesthesia with phenobarbital (30 mg/kg), followed by bilateral ACL reconstruction. The knee joint was accessed via a medial parapatellar approach through a midline longitudinal incision. After lateral patellar dislocation, the normal ACL was excised at femoral and tibial origins. Femoral and tibial tunnels were created with a 3.0 mm diameter drill-bit based on the footprints of the normal ACL. The long digital extensor tendon (2 mm in diameter and 3 cm in length) was harvested as the tendon graft. Both graft ends were braided with Dexon 3–0 suture and passed through the drilling holes, then graft ends were fixed to the tunnel exits with sutures tied over the neighboring periosteum. The PLA, PLA/HA, or PLA/HA loaded MSCs screw-like scaffolds were then pressed into the femoral tunnel of each rabbit (Fig. 2). The rabbits were allowed free cage movement after the operation with intramuscular injection of penicillin (800,000 U) once daily for 3 consecutive days. The rabbits were sacrificed at 4 and 12 weeks (12 rabbits total, 6 at each time point) for magnetic resonance imagery (MRI), micro-computed tomography (micro-CT), and histological examinations.

### MRI Examination

All specimens were examined with a 7.0 T magnetic resonance imaging (MRI) system for small animals (Agilent VnmrJ 3.1, Agilent Technologies, USA) to observe graft and implant status in the transverse, coronal, and sagittal sections. The scan parameters were: number of sections = 20, section thickness = 1.00 mm, TR/TE = 600 ms/8 ms, acquisition matrix = 384 × 192, and FOV = 40 mm × 40 mm.

### Micro-CT Analysis

**Figure 3:**
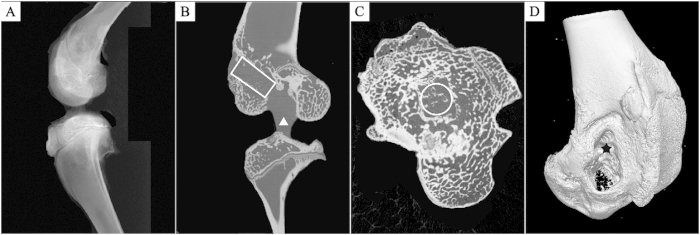
The sketch map of micro-CT evaluations. (**A**) Gross observation of the vertical plane of the rabbit knee joint. (**B**) The vertical plane of the axis of the femoral bone tunnel in a sagittal view of the micro-CT image, the region of interest (ROI) was shown with new bone within the bone tunnel (white rectangle) and the tendon graft can be observed (white triangle). (**C**) The cross section of the axis of the femoral bone tunnel of the micro-CT image, ROI was shown with new bone within the bone tunnel (white circle). (**D**) The external aperture of the femoral bone tunnel in a 3D reconstruction micro-CT image (black star), new bone growth can be seen within the bone tunnel.

Micro-CT measurement was performed using a micro-CT system (vivaCT100, Scanco Medical, Switzerland; 80 kVp, 80 mA) for quantifying mineralized tissue ingrowth inside the bone tunnel (n = 5). Each specimen was scanned perpendicular to the long bone axis covering the entrance and exit of the femoral tunnel. To determine the amount and quality of the newly formed mineralized tissue over time, a 3-mm circular region of interest (ROI) inside the bone tunnel was chosen and three-dimensionally reconstructed using MicView software (Fig. 3).

### Histological Analysis

Samples were prepared for histological analysis, without decalcification, at each respective analysis point. The samples were fixed in 4% paraformaldehyde solution for 7 days, dehydrated with graded alcohols (70, 75, 80, 85, 90, 95, 100%), cleaned with toluene, and embedded in MMA. The embedded specimens were then sectioned in the anterioreposterior direction and parallel to the longitudinal axis of the long bone by saw microtome (SP1600, Leica, Germany). Finally, the sections were grinded and polished to 40–50 mm (Exakt-Micro-Grindin System, Leica, Germany) and stained with Von-Gieson to examine the new bone and the healing at the tendon graft-bone tunnel interface.

### Statistical Analysis

All numerical data were expressed as the mean value ± standard deviation (SD). Statistical analysis was performed by one-way analysis of variance (AVOVA), and *p* < 0.05 was considered to be significant.

## Results

### Physical Properties of PLA Screw-like Scaffold

**Figure 4:**
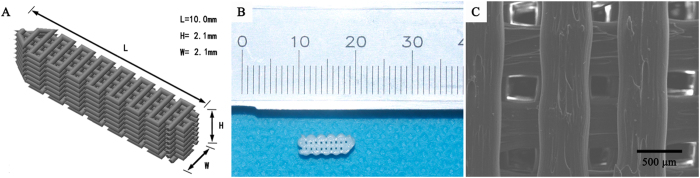
Physical properties of the PLA screw-like scaffold. (**A**) 3D view of the theoretical designed PLA screw-like scaffold structure. (**B**) The prepared PLA screw-like scaffold. (**C**) The SEM image of the PLA scaffold surface with well-defined orthogonal structure.

The PLA screw-like scaffold was designed to generate the theoretical structure discussed above (Fig. 4). We confirmed through SEM that the scaffold possessed well-defined orthogonal structure with macropores around 290 ± 16 μm in size. Scaffold porosity was measured with the Archimedes method at 42 ± 5%, which was in accordance with our design.

### Physicochemical HA Characterization

**Figure 5:**
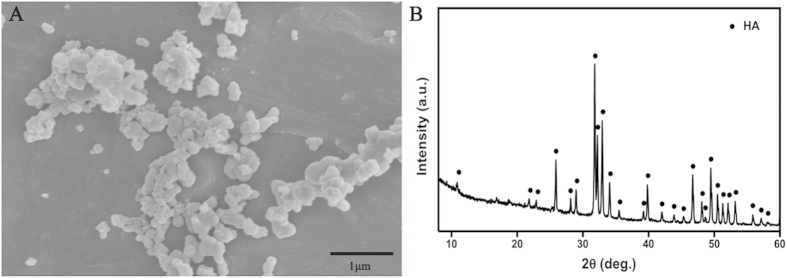
Characterization of synthesized hydroxyapatite (HA). (**A**) The SEM image of HA crystals. (**B**) The XRD spectrum of HA crystals.

The synthesized HA were characterized after chemical precipitation synthesis using Ca(NO_3_)_2_·4 H_2_O and (NH_4_)_2_HPO_4_ as P and Ca precursors, respectively. SEM observation (Fig. 5A) showed HA crystals around 200 nm in diameter. Figure 5B shows the XRD patterns of the synthesized HA composites. In the spectrum, all diffraction peaks as-assigned are in agreement with the standard HA (JCPDS09-0432). XRD analysis also revealed that HA exhibited sharp diffraction peaks, indicating high crystallinity, which demonstrated that the HA powders had been synthesized successfully.

### PLA/HA Scaffold Characterization

**Figure 6:**
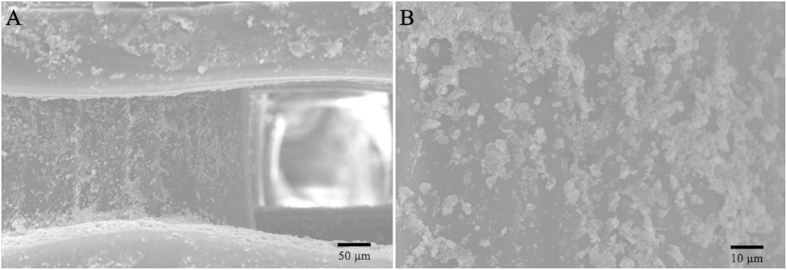
SEM images of the PLA/HA scaffold. (**A**) The modification of HA on the 3D structure of the PLA scaffold. (**B**) The closer observation of the HA modification on the PLA surface.

The surface modification on the PLA/HA scaffold was directly observed using SEM. Figure 6 shows that HA crystals were evenly distributed on the PLA structure – not only on the surface of the scaffold, but also in the deeper structure around macropores in the scaffold due to vacuum conditions during modification.

### Cell Morphology and Viability

**Figure 7:**
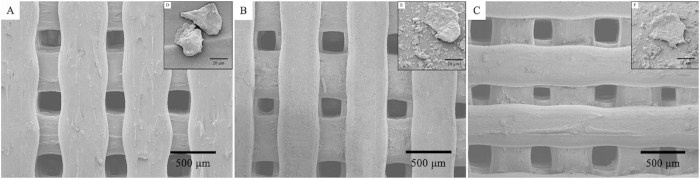
SEM images of the MSCs seeded on (**A**) PLA scaffold, (**B**) PLA/HA scaffold and (**C**) suspended in Pluronic F-127 solution on PLA/HA scaffold for 48 hours. The photographs (**D–F**) are from closer observation of MSCs, respectively.

Cell morphology was investigated using SEM to obtain qualitative information for the three groups (MSCs seeded on the PLA scaffold, PLA/HA scaffold, and MSCs suspended in Pluronic F-127 solution seeded on PLA/HA scaffold). The SEM images (Fig. 7) showed that the MSCs suspended in Pluronic F-127 solution on PLA/HA scaffold were polygonal or widespread in form, with fine filopodia and abundant surface folds. By contrast, round or spherical cells with fewer filopodia were observed in PLA than those in PLA/HA groups.

**Figure 8:**
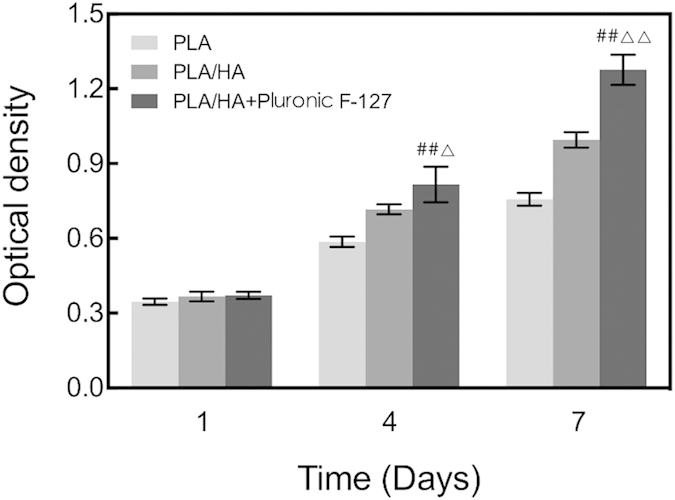
CCK-8 assay for MSCs proliferation on PLA scaffold, PLA/HA scaffold, and suspended in Pluronic F-127 solution on PLA/HA scaffold at 1day, 4 days and 7 days. ^##^vs. PLA scaffold, p < 0.01; ^△^vs. PLA/HA scaffold, p < 0.05; ^△△^vs. PLA/HA scaffold, p < 0.01; n = 5.

The viability of the MSCs in all three groups were examined by CCK-8 assay after 1, 4, and 7 days of incubation (Fig. 8). MSC viability in the three groups was similar at Day 1 (*p* > 0.05). At Day 4, the viability of MSCs suspended in Pluronic F-127 on PLA/HA scaffold was higher than those seeded on PLA scaffold (*p* < 0.01) or on PLA/HA scaffold (*p* < 0.05). At Day 7, MSCs suspended in Pluronic F-127 on PLA/HA scaffold demonstrated the highest viability compared to that of MSCs seeded on PLA scaffold (*p* < 0.01) or on PLA/HA scaffold (*p* < 0.01).

### Real-time PCR for Col I, OCN, Sp7, and Runx2 Gene Expression

**Figure 9:**
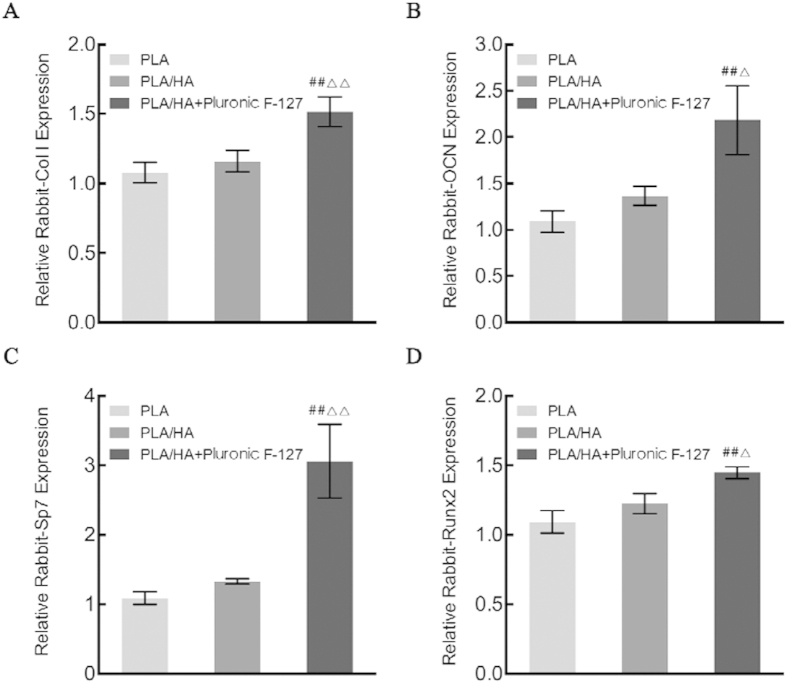
RT-PCR analysis for the osteoblast phenotypic marker genes: the level of (**A**) Col I, (**B**) OCN, (**C**) Sp7 and (**D**) Runx2 mRNA in MSCs seeded on PLA scaffold, PLA/HA scaffold, and suspended in Pluronic F-127 solution on PLA/HA scaffold at 7 days. ^##^vs. PLA scaffold, p < 0.01; ^△^vs. PLA/HA scaffold, p < 0.05; ^△△^vs. PLA/HA scaffold, p < 0.01.

As shown in Fig. 9, real-time PCR was carried out to detect gene expression during osteogenic differentiation. MSCs treated by Pluronic F-127 on PLA/HA scaffold exhibited the highest gene expressions of Col I, OCN, Sp7, and Runx2 among the three groups at Day 7 (*p* < 0.05).

### MRI Examination

**Figure 10:**
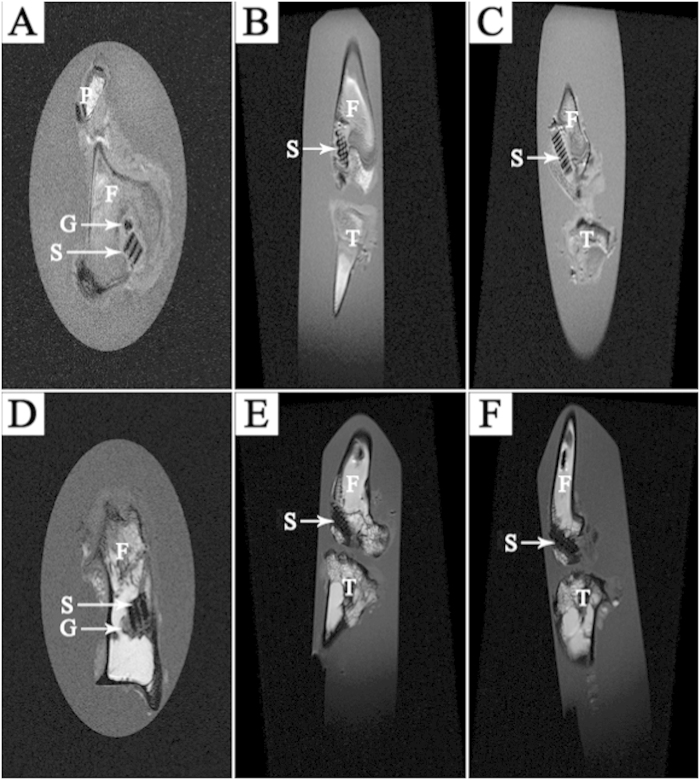
Images of MRI examination in MSCs groups: (**A**) transverse, (**B**) coronal and (**C**) sagittal section at 4 weeks; (**D**) transverse, (**E**) coronal and (**F**) sagittal section at 12 weeks. (G: Graft; S: Screw-like scaffold; F: Femur; T: Tibia; P: Patella).

According to the MRI examination, all screw-like scaffolds were correctly positioned in the bone tunnel without any breakage or major complications. The transverse, coronal, and sagittal slices all showed the well-defined orthogonal structure of the screw-like scaffold, with clearly observable macropores. The tendon graft was easily found within the bone tunnel in the transverse section. Given no statistical analysis was conducted for this examination, MSC groups are shown as an example of MRI examination both at 4 and 12 weeks (Fig. 10). Compared to the MRI sections at 4 weeks, the MRI sections at 12 weeks displayed closer combination between the bone tunnel and the screw-like scaffold.

### Micro-CT Analysis

**Figure 11:**
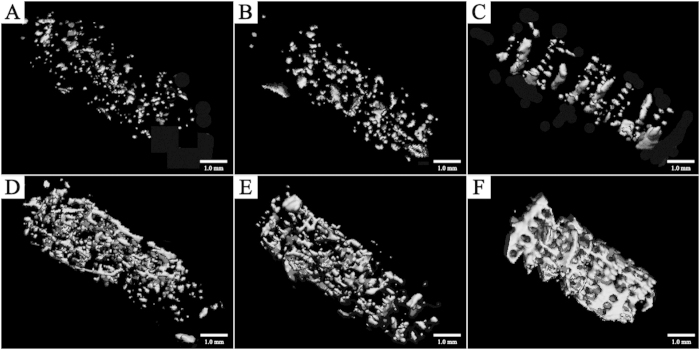
3D reconstruction micro-CT images of new bone formation within the femoral bone tunnel. At 4 weeks, the volume of new bone growth in the MSCs group (**C**) was similar to that in the PLA/HA group (**B**), but more than that in the PLA group (**A**); at 12 weeks, the new bone was well distributed and interconnected in the MSCs group (**F**) and the volume of its new bone formation was more than that in the PLA/HA group (**E**) which is similar to that in the PLA group (**D**).

New mineralized tissue in bone tunnels was measured by micro-CT analysis. As shown in Tables 2 and 3, the MSCs groups exhibited more mineralized tissue formation than the other two groups both at Week 4 and Week 12 with higher BV/TV, Tb.N, and Tb.Th, and lower Tb.Sp. The 3D reconstructed measurement (Fig. 11) yielded the same results for MSCs groups at Week 4 and Week 12 as the above micro-CT evaluation. The new mineralized tissue was well-distributed and interconnected gradually in the bone tunnel due to the infiltration and growth of new bone within macropores and interconnected pores of the screw-like scaffold. Notably, the interconnected mineralized tissue of MSCs groups at Week 12 demonstrated a shape similar to the screw-like scaffold (Fig. 11F); this outcome demonstrates that a 3D-printed screw-like scaffold can match the speed of new bone formation around a tendon graft and minimize the effect of slow-degrading PLA impeding new bone growth.

### Histological Examination

**Figure 12:**
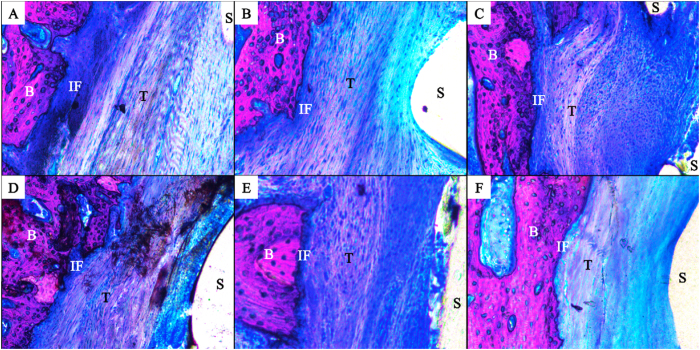
Histology images in the rabbit ACL reconstruction model. At 4 weeks, there was full of fibrous tissue with less new bone formation was found at the interface tissue in the PLA group (**A**). The interface tissue contains more chondrocytes and cartilage matrix in the MSCs group (**C**) than in the PLA/HA group (**B**); At 12 weeks, the spaces between the tendon graft and the bone tunnel were narrower in all three groups (**D–F**). Compared with the PLA group (**D**) and PLA/HA group (**E**), the tendon graft side was in intimate contact with the new bone and increased collagen fiber continuity between the new bone and the tendon in the MSCs group (**F**). (B: Bone; IF: Interface; T: Tendon graft; S: Screw-like scaffold; Von-Gieson stain, original magnification ×100).

Tendon graft healing within the bone tunnel was observed by histological examination. After 4 weeks, the interface (IF) tissue contained more chondrocytes and cartilage matrix in the MSCs group (Fig. 12C) than in the PLA/HA group (Fig. 12B); the PLA group was full of fibrous tissue, with less new bone formation (Fig. 12A). After 12 weeks, the spaces between the tendon graft and bone tunnel were narrower in all three groups. Compared to the PLA group (Fig. 12D) and PLA/HA group (Fig. 12E), MSCs group (Fig. 12F) had tendon grafts in much closer contact with the new bone, and showed increased collagen fiber continuity between the new bone and the tendon.

## Discussion

3D printing technology has changed the daily lives of many over the course of its development, particularly after the D3DP became a popular tool for fabricating personalized consumer products such as electrical components and bicycle parts easily, economically, and even in the home^26^. The rapid development of clinical medicine, which has increased alongside growing interest in the concept of translational medicine, involves clinics relying not only on medicine itself but also on a combination of engineering, biomaterial, and informatics technologies^27^. The doctor, the backbone of a clinic, can provide more personalized and effective medical services by mastering fundamental knowledge in addition to new techniques, skills, and tools. The emergence of new 3D printing technology, especially the D3DP industry, essentially builds a bridge integrating medical knowledge with advanced techniques for actualizing innovative pursuits of doctors and specialists^4^,^28^,^29^.

In this study, we used the fabrication of surgical implants to demonstrate that customized and surgical implants can be fabricated at the clinic (fab@clinic) successfully and economically with a D3DP. The reason we assert that fab@clinic can be actualized with D3DPs is three-fold: 1) the D3DP will become the personal 3D printer (P3DP) gradually as patent protection expires and open source hardware develops^30^, 2) because D3DP operation is remarkably simple, doctors, surgeons, and patients can readily reap the benefits of highly customizable material, and 3) the cost of consumables such as PLA is quite low (10–50 dollars/kg for industrial grade and about 150–500 dollars/kg for medical grade), fitting into most budgets as easily as an ink-jet printer and its necessary supplies. In our study, the cost of fabricating an implant applied in a rabbit ACL reconstruction model was only about 50 cents with industrial grade PLA (Table 4), and it will be no more than 10 dollars using medical grade PLA in future clinical application.

Although all facilities in our study were very simple and cost-effective, we successfully combined them with a D3DP to improve our surgical experiment. The successful application of PLA filament, the most common printing material used in D3DPs, to fabricate surgical implants firmly demonstrated the replicability of the fab@clinic method proposed here. PLA is a good biomaterial polymer for ensuring that screws achieve stable fixation of grafts in arthroscopic surgery, and it has favorable biocompatibility for cell adhesion and ACL fibroblast proliferation^31^. That said, the slow degradation rate of PLA screws impedes the quantity and volume of new bone formation in the bone tunnel. The D3DP effectively overcame this disadvantage by introducing well-arranged macroporous structure followed by HA surface modification^32^,^33^ into our screw-like scaffold, not only enhancing new bone ingrowth but also the proliferation and migration of MSCs and osteoblasts with excellent vascularization^34^. The SEM observation results confirmed that macropores and interconnected pores were arranged in regular patterns with orthogonal structure and size corresponding to the ideal theoretical value. New bone tissue formation *in vivo* was distributed within the PLA scaffold as expected, confirmed through Micro-CT analysis.

Well-designed scaffolds can be easily manufactured with a D3DP and open source hardware to fit the 3D culture environment for MSCs, which was thought to be the most suitable cell type for ligament tissue engineering compared against ACL fibroblast and skin fibroblast^35^. Therefore, MSCs were used in this study as the cell source. As expected, the MSCs group exhibited the most new bone formation within bone tunnel out of all groups through *in vivo* testing, and histological examination indicated that new bone formation was increased at the interface between the bone tunnel and tendon graft after 12 weeks – in effect, the MSC-treated group achieved ideal bone-tendon healing compared to the other groups, which was similar to results of previous studies on MSCs^35^,^36^. The results of this study, to this effect, pose another approach to optimizing tissue engineering technology through the use of low-cost D3DPs.

**Figure 13:**
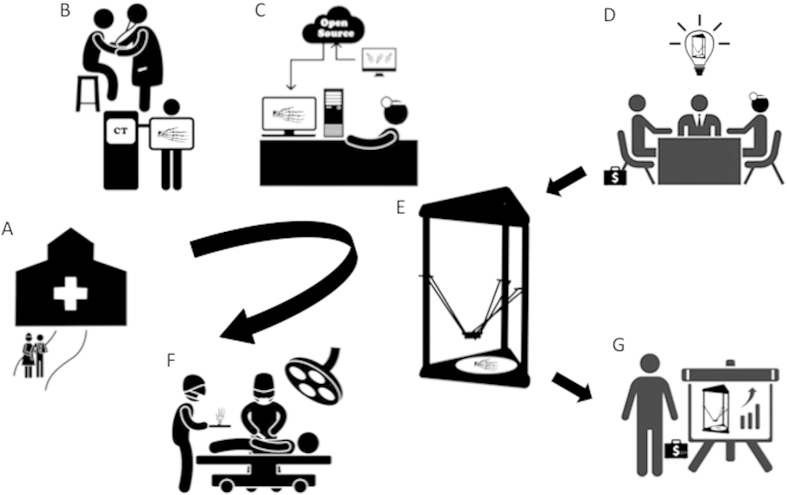
The concept map of the fab@clinic D3DP. (**A**) Patients who suffer trauma went to the hospital for treatment. (**B**) Patients get consultation in hospital and do radiology examination. (**C**) With the help of open source online and radiologic data, surgeons design and reconstruct customized surgical implants in the office. (**D**) Surgeons, company mangers and sellers get together to develop the fab@clinic D3DP. (**E**) The customized implants are fabricated with the fab@clinic D3DP in the surgeons’ office. (**F**) The customized implants are implanted during the operation. (**G**) Since fab@clinic D3DPs are widely applied in surgery, the sales volume will increase rapidly to achieve a big market and create considerable economic value. Figure 13 was drawn by Miao Sun.

The 3D printing industry is developing at an extraordinary pace^37^, with a variety of novel components containing electric, flexible, and water-soluble materials^38^ springing up constantly. More biocompatible, implantable printing materials combined with other growth factors and cell sources will certainly emerge, as well, to meet the increasing demand for medical innovations^39^. To keep up, doctors should become adept at utilizing D3DPs and other new technologies to solve problems and mediate difficulties; D3DPs are especially helpful tools, as they are conveniently operated and low in cost. As a result, fab@clinic alternatives can reach a larger market and the resources and skills required for doctors and specialists to realize new ideas can be made more readily available, setting off a positive chain reaction of innovation, application, and commercialization within clinical medicine in the near future (Fig. 13).

This study was not without limitations. First, the screw-like scaffold as-designed and the rabbit model may not fully mimic human physiological conditions. Although no graft rupture or screw-like scaffold cracking in our study was observed via MRI analysis, the porous structure applied in bioabsorbable screws in ACL reconstruction must be very well-designed to ensure essential mechanical strength for patients. Additionally, we did not label or track MSCs within the screw-like scaffold – future research should do so in order to better examine the mechanism of MSCs within the screw-like scaffold during tendon-bone healing.

This study used a D3DP to fabricate screw-like scaffolds, combined with MSCs, to fix tendon grafts and promote tendon graft healing within the bone tunnel in a rabbit ACL reconstruction model. Results showed that tendon graft healing within the bone tunnel was best when the PLA/HA scaffold was loaded with MSCs and implanted into the bone tunnel, confirmed by a high level of bone ingrowth and favorable bone-graft interface formation. We hope that this study sets a good example for fab@clinic using an easily operated, low-cost D3DP printer.

## Additional Information

**How to cite this article**: Liu, A. *et al.* 3D Printing Surgical Implants at the clinic: A Experimental Study on Anterior Cruciate Ligament Reconstruction. *Sci. Rep.* **6**, 21704; doi: 10.1038/srep21704 (2016).

## Figures and Tables

**Table 1:** The parameters of primers utilized for detecting osteogenetic gene expression.

Gene	Direction	Primer sequence (5′–3′)
Col I	Forward	GCG GTG GTT ACG ACT TTG GTT
	Reverse	AGT GAG GAG GGT CTC AAT CTG
OCN	Forward	GGC TCA GCC TTC GTG TCC AA
	Reverse	CCC TGC CCG TCG ATC AGT T
SP7	Forward	GGC ACG AAG AAG CCA TAC TCT GT
	Reverse	GGG AAA AGG CCG GGT AGT CAT
RUNX2	Forward	CCC AAG CAT TTC ATC CCT CAC T
	Reverse	CAT ACC GAG GGA CAT GCC TGA
GADPH	Forward	TCA CCA TCT TCC AGG AGC GA
	Reverse	CAC AAT GCC GAA GTG GTC GT

**Table 2:** Micro-CT evaluation at 4 weeks.

Items	PLA (n = 5)	PLA/HA (n = 5)	MSCs (n = 5)
BV/TV (%)	1.466 ± 0.185	4.672 ± 0.410^##^	6.148 ± 0.626^##△△^
Tb. N (1/mm)	0.825 ± 0.061	1.016 ± 0.057^##^	1.210 ± 0.077^##△△^
Tb. Th (mm)	0.120 ± 0.013	0.137 ± 0.007	0.142 ± 0.009^#^
Tb. Sp (mm)	1.127 ± 0.070	0.951 ± 0.077^##^	0.845 ± 0.054^##^

BV, bone volume; TV, total volume; Tb. N, trabecular number; Tb. Th, trabecular thickness; Tb. Sp, trabecular separation. ^#^vs. PLA, p < 0.05; ^##^vs. PLA, p < 0.01; ^△△^vs. PLA/HA, p < 0.01.

**Table 3:** Micro-CT evaluation at 12 weeks.

Items	PLA (n = 5)	PLA/HA (n = 5)	MSCs (n = 5)
BV/TV (%)	12.586 ± 1.882	14.416 ± 3.458	27.456 ± 2.282^##△△^
Tb. N (1/mm)	1.439 ± 0.084	1.503 ± 0.067	1.796 ± 0.198^##△^
Tb. Th (mm)	0.177 ± 0.020	0.179 ± 0.012	0.267 ± 0.031^##△△^
Tb. Sp (mm)	0.815 ± 0.062	0.780 ± 0.045	0.570 ± 0.089^##△△^

BV, bone volume; TV, total volume; Tb. N, trabecular number; Tb. Th, trabecular thickness; Tb. Sp, trabecular separation. ^##^ vs. PLA, p < 0.01; ^△^ vs. PLA/HA, p < 0.05; ^△△^ vs. PLA/HA, p < 0.01.

**Table 4:** The fabrication cost of a PLA screw-like scaffold with a low-cost 3D printer in a rabbit ACL reconstruction model.

Items	Amount	Unit price	Cost
PLA filament	1g	$31/kg	$0.031
Power consumption	≈0.005 kw·h	$0.24/ kw·h	≈ $0.0012
Labor cost	3 min	$5/h	$0.25
Others (machine loss, etc.)			$0.2
Total cost			≈ $0.5
